# Predictors, Outcomes, and Statistical Solutions of Missing Cases in Web-Based Psychotherapy: Methodological Replication and Elaboration Study

**DOI:** 10.2196/22700

**Published:** 2021-02-05

**Authors:** Eyal Karin, Monique Frances Crane, Blake Farran Dear, Olav Nielssen, Gillian Ziona Heller, Rony Kayrouz, Nickolai Titov

**Affiliations:** 1 Department of Psychology Macquarie University MindSpot Clinic Macquarie Park Australia; 2 Department of Psychology Macquarie University eCentreClinic Sydney Australia; 3 Department of Psychology Macquarie University MindSpot Clinic Sydney Australia; 4 Department of Statistics Macquarie University Sydney Australia

**Keywords:** psychotherapy, treatment adherence and compliance, missing data, treatment evaluation, statistical bias

## Abstract

**Background:**

Missing cases present a challenge to our ability to evaluate the effects of web-based psychotherapy trials. As missing cases are often lost to follow-up, less is known about their characteristics, their likely clinical outcomes, or the likely effect of the treatment being trialed.

**Objective:**

The aim of this study is to explore the characteristics of missing cases, their likely treatment outcomes, and the ability of different statistical models to approximate missing posttreatment data.

**Methods:**

A sample of internet-delivered cognitive behavioral therapy participants in routine care (n=6701, with 36.26% missing cases at posttreatment) was used to identify predictors of dropping out of treatment and predictors that moderated clinical outcomes, such as symptoms of psychological distress, anxiety, and depression. These variables were then incorporated into a range of statistical models that approximated replacement outcomes for missing cases, and the results were compared using sensitivity and cross-validation analyses.

**Results:**

Treatment adherence, as measured by the rate of progress of an individual through the treatment modules, and higher pretreatment symptom scores were identified as the dominant predictors of missing cases probability (Nagelkerke *R*^2^=60.8%) and the rate of symptom change. Low treatment adherence, in particular, was associated with increased odds of presenting as missing cases during posttreatment assessment (eg, odds ratio 161.1:1) and, at the same time, attenuated the rate of symptom change across anxiety (up to 28% of the total symptom with 48% reduction effect), depression (up to 41% of the total with 48% symptom reduction effect), and psychological distress symptom outcomes (up to 52% of the total with 37% symptom reduction effect) at the end of the 8-week window. Reflecting this pattern of results, statistical replacement methods that overlooked the features of treatment adherence and baseline severity underestimated missing case symptom outcomes by as much as 39% at posttreatment.

**Conclusions:**

The treatment outcomes of the cases that were missing at posttreatment were distinct from those of the remaining observed sample. Thus, overlooking the features of missing cases is likely to result in an inaccurate estimate of the effect of treatment.

## Introduction

### Background

The ability to evaluate the effect of psychotherapy often depends on the measurement of outcomes before-and-after an intervention. However, many participants are unable to complete measurement questionnaires and become missing cases, thus threatening the validity of conclusions drawn from trials. Missing cases are frequently reported in psychotherapy trials [1,2] and pose a risk to the validity of the evidence base for some treatments [3,4]. Overlooking the causes and outcomes of missing cases can lead to systematic measurement bias and misrepresentation of treatment outcomes and, therefore, risks compromising the validity of clinical research [5,6]. For this reason, careful analysis of the effect of missing cases is now considered an important part of the process of measuring and reporting clinical evidence [3].

Although the importance of handling missing cases is well understood [3,7], accounting for the outcomes of missing cases is a challenging task, as researchers can never verify whether the replacement values they generate accurately captured patient outcomes. Thus, researchers must rely on statistical approximation and the assumption that any replacement outcomes are suitable [8].

A key requirement for handling missing data is to ensure that the outcomes of missing cases are represented within statistical analyses [8]; typically, this involves using a statistical solution that generates replacement values for missing cases [5,8,9]. Researchers rely on statistical methods that explore the characteristics of missing cases to determine whether a statistical solution is suitable for missing cases and whether these features could also be associated with distinct clinical outcomes. This is typically achieved through analyses that identify variables that predict both the probability that participants will become missing cases and the clinical outcome of such missing cases [4,8,10]. Identifying such variables enables researchers to generate replacement scores that are likely to capture the outcomes of treatment for missing cases [7,10]. For example, if older age is associated with a decreased probability of becoming a missing case and an increased rate of symptom change, a statistical model that can adjust for participants’ age will be considered to create replacement outcomes that are more accurate and representative of the effects of treatment than models that overlook age. In statistical terms, variables that predict both the likelihood of becoming a missing case and the outcome of missing cases are known as mechanisms of nonignorable missing cases [6,10,11].

Although statistical models that incorporate replacement values for missing cases have been in use for decades [7,8,12], relatively few published studies have reported the characteristics of missing cases in psychotherapy trials or research that identified nonignorable mechanisms of noncompletion that might influence the reported outcomes [2,13]. This gap in methodological research may result from (1) the limited knowledge about missing cases and the patient features that may generalize across clinical trials [2] and (2) the scarcity of large and comparable treatment samples that are statistically powered to explore nonignorable mechanisms of noncompletion.

Preliminary evidence from trials of internet-delivered cognitive behavioral therapy (iCBT) suggests that common patient variables, such as treatment completion and baseline depressive symptom severity, were the main predictors of both the likelihood of patients dropping out of treatment and moderating the clinical effect [2,4]. These findings suggested that (1) the symptom outcomes of missing cases were not comparable with the patients that provided their data following treatment and (2) missing cases can be characterized through key features that shape the likelihood of a case to present as missing during posttreatment assessment. In particular, minimal treatment adherence, as measured by the partial progress of an individual through the treatment modules, was associated with increased odds of presenting as a missing case during posttreatment assessment (eg, odds ratio 70.6, 95% CI 34.5 to 145.1) and a lower rate of symptom change (eg, 21% for low treatment adherence vs 49% for high adherence) [4]. Without accounting for these variables, web-based psychotherapy researchers risk overlooking a systematic pattern of worse treatment outcomes for missing cases and generating estimates of treatment effects that are unrealistically optimistic. However, the evidence from this study regarding the effect of missing cases in internet-delivered psychotherapy is limited to a single study that focused on symptoms of depression using data from a highly controlled clinical trial with high participant retention (87%) [4]. Replicating this study in an additional therapeutic context and within additional clinical outcomes is needed before conclusions can be drawn regarding the characteristics and effect of missing cases in internet-delivered psychotherapy and the appropriate statistical methods for handling missing cases.

### Objectives

The main aim of this study is to examine the characteristics and possible clinical outcomes of missing cases in a large sample in routine care and compare different statistical methods for estimating those outcomes. This study examined the outcomes of a large sample of patients enrolled in treatment courses provided by an established digital mental health service (DMHS) offering internet psychotherapy based on cognitive behavior therapy (n=6701), in which the patients were administered validated self-report questionnaires to measure symptoms of depression, anxiety, and psychological distress at baseline, at intervals during treatment, and at follow-up. It was hypothesized that (1) lower treatment completion and increased baseline depressive symptoms would predict both increased likelihood of noncompletion and higher symptoms of depression posttreatment and that (2) statistical models that account for these features will result in higher posttreatment symptom replacement scores compared with the statistical models that assume missing cases occur as a random event.

## Methods

### The Sample

This study examined the outcome of routine care provided by Australian National DMHS, the MindSpot Clinic [14]. All participants provided consent for their deidentified data to be used in evaluation and quality improvement activities. Approval for this research was provided by the Macquarie University Human Research Ethics Committee. Further information about the sample, the course content and delivery protocols, and the outcomes of the iCBT can be found in a study by Titov et al [15]. The standardized nature of clinical engagement and treatment delivery in iCBT reduces the likelihood that differences in outcomes are because of different approaches of therapists.

The 6701 participants who commenced treatment during a 30-month period completed self-report symptom scales and provided other information pretreatment and completed symptom scales midtreatment (surveyed at Week 4), posttreatment (Week 8), and at follow-up (Week 20).

In this study, emphasis was on the prediction of posttreatment symptom outcomes, where posttreatment was considered the main time point for evaluating the effects of treatment [15]. From the participants who initiated treatment, 63.7% (4271/6701) of the sample provided data posttreatment, with 36% (2430/6701) considered to be missing cases as individuals who did not comply with weekly email and telephone prompts to complete a posttreatment evaluation assessment. For cross-replication analysis, the sample was randomly allocated into 5 subgroups, each with more than 1340 participants pretreatment and more than 840 completed measurements posttreatment. Tables 1 and 2 collate the demographic information of the samples, including chi-square values, to confirm adequate randomization.

**Table 1:** Randomization of cross-validation samples and participant characteristics (N=6701).

Sample		Available sample at pretreatment, n (%)	Available sample at posttreatment, n (%)	Randomization test	
				Chi-square (*df*)	*P* value
**Total sample**		6701 (100)	4271 (64)	0.01 (4)	.99
	Replication sample 1	1341 (20.01)	842 (62.79)	N/A^a^	N/A
	Replication sample 2	1340 (20.00)	846 (63.13)	N/A	N/A
	Replication sample 3	1340 (20.00)	843 (62.91)	N/A	N/A
	Replication sample 4	1340 (20.00)	846 (63.13)	N/A	N/A
	Replication sample 5	1340 (20.00)	848 (63.28)	N/A	N/A

^a^N/A: not applicable (redundant parameter).

**Table 2:** Sample demographics.

Variable	Value	Randomization test	
		Chi-square (*df*)	*P* value
Age (years), mean (SD)	37.57 (10.9)	3.8 (1)	.44
Completed 1/5 modules, n (%)	513 (7.66)	7.5 (4)	.96
Completed 2/5 modules, n (%)	715 (10.67)	N/A^a^	N/A
Completed 3/5 modules, n (%)	718 (10.71)	N/A	N/A
Completed 4/5 modules, n (%)	653 (9.74)	N/A	N/A
Completed 5/5 modules, n (%)	4102 (61.21)	N/A	N/A
In a relationship, n (%)	4458 (66.53)	0.6 (1)	.97
Employment (employed), n (%)	4908 (73.24)	0.8 (1)	.94
Education (tertiary), n (%)	3239 (48.34)	4.0 (1)	.41
Gender (female), n (%)	4866 (48.34)	6.8 (1)	.15
Comorbidity (GAD-7^b^ ≤8 and PHQ-9^c^ ≤10), n (%)	3437 (51.29)	3.0 (1)	.56

^a^N/A: not applicable (redundant parameter).

^b^GAD-7: generalized anxiety disorder-7 item scale.

^c^PHQ-9: patient health questionnaire-9.

### Intervention

The participants enrolled in the Wellbeing Course [15], a 5-lesson course delivered over 8 weeks to patients experiencing depression and anxiety. The lessons covered (1) the cognitive behavioral model and symptom identification, (2) thought monitoring and challenging, (3) de-arousal strategies and pleasant activity scheduling, (4) graduated exposure, and (5) relapse prevention. Additional material included downloaded lesson summaries, patient stories, and a range of resources, for example, improved sleep, problem solving, and communication. Each of the lessons provided homework assignments to assist participants in learning and applying the skills described in the lessons to their everyday lives.

### Measures

The primary outcome measures for this study were standardized symptom scales for anxiety, depression, and psychological distress.

### Patient Health Questionnaire-9

Patient Health Questionnaire-9 (PHQ-9) is a 9-item measure of depressive symptoms. Total scores range from 0 to 27 with higher scores indicating more severe depressive symptoms. PHQ-9 has demonstrated excellent reliability and validity in previous studies [16,17] and high internal reliability (Cronbach α=.848) and stability over time (assessment to pretreatment intraclass correlation=.72) within this sample.

### Generalized Anxiety Disorder Scale-7 Item

Generalized Anxiety Disorder Scale-7 Item (GAD-7) is a 7-item measure of generalized anxiety. Total scores range from 0 to 21, with higher scores indicating more severe symptoms of anxiety. GAD-7 has shown excellent reliability and validity in previous studies [17,18] and high internal reliability (Cronbach α=.85) and stability over time (assessment to pretreatment intraclass correlation=.74) within this sample.

### Kessler 10 Item

Kessler 10 Item (K-10) is a widely used 10-item measure of psychological distress. The scale has demonstrated adequate reliability and validity in previous studies [17,19] and within this sample (Cronbach α=.83; intraclass correlation=.71). Total scores range from 10 to 50 with higher scores indicating greater levels of psychological distress. The 10 to 50 score range was converted into a 0 to 40 range within the analysis of longitudinal symptom change.

The following measures were also included as possible independent variables or predictors that might predict clinical trajectory through treatment and noncompletion.

### Comorbidity

Individuals were considered to have comorbidity if they demonstrated scores of both anxiety and depression above predetermined clinical thresholds (GAD-7 ≥8 and PHQ-9 10 at baseline [17]).

### Demographic Measures

This included age (in years at the start of treatment), gender, relationship status, pretreatment symptom scores, pretreatment anxiety scores, and educational attainment (Tables 3 and 4).

**Table 3:** Univariate missing cases probability models of the total sample (N=6701).

Variable considered			Variable test *P* value	Time* predictor odds ratio (95% CI)	Variance explained (*R*^2^)	RRI^a^ % missing, odds ratio (95% CI)	Model sensitivity (true positive, %)	Model specificity (true negative, %)	Overall model accuracy, %	AUROC (95% CI)^b^
Sample average			<.001	0.566 (.539 to .595)	N/A^c^	36 (35 to 37)	N/A	N/A	N/A	N/A
**Demographic**										
	Age (% per year)		<.001	0.967 (.963 to .972)	3.80	–1 (–1.1 to –1.1)	62.60	53.00	56.50	0.603 (0.589 to 0.617)
	**Gender**									
		Female	.003	1.188 (1.06 to 1.330)	0.20	37 (36 to 39)	74.80	28.60	45.30	0.517 (0.503 to 0.531)
		Male	N/A	N/A	N/A	33 (31 to 35)	N/A	N/A	N/A	N/A
	**Employment status**									
		At least some employment	.62	0.972 (.868 to 1.088)	0.00	36 (35 to 37)	73.10	26.90	56.80	0.503 (0.488 to 0.517)
		Otherwise	N/A	N/A	N/A	37 (34 to 39)	N/A	N/A	N/A	N/A
	**Relationship status**									
		In a relationship	0.01	0.876 (.789 to .974)	0.10	35 (34 to 37)	67.80	64.80	56.80	0.515 (0.500 to 0.529)
		Otherwise	N/A	N/A	N/A	38 (36 to 40)	N/A	N/A	N/A	N/A
	**Education level**									
		Tertiary education	<.001	0.736 (.665 to .813)	0.70	32 (31 to 34)	56.50	51.30	53.10	0.538 (0.524 to 0.553)
		Otherwise	N/A	N/A	N/A	40 (38 to 41)	N/A	N/A	N/A	N/A
**Initial severity**										
		Baseline anxiety symptoms (% per GAD-7^d^ point)	<.001	1.024 (1.014 to 1.034)	0.50	0.7 (0.7 to 0.72)	53.10	54.80	51.00	0.535 (0.521 to 0.549)
		Baseline depression symptoms (% per PHQ-9^e^ point)	<.001	1.037 (1.028 to 1.046)	1.40	1.4 (1.4 to 1.44)	56.10	53.10	55.00	0.562 (0.548 to 0.576)
		Baseline psychological distress (% per K-10^f^ point)	<.001	1.033 (1.026 to 1.040)	1.90	1.1 (1.1 to 1.08)	54.80	55.50	55.00	0.571 (0.557 to 0.585)
		Comorbidity at baseline: (PHQ-9 ≥10 and GAD-7 ≥8)	<.001	0.718 (0.649 to 0.793)	0.90	40 (38 to 42)	N/A	N/A	N/A	0.541 (0.556 to 0.527)
		None	N/A	N/A	N/A	32 (31 to 34)	N/A	N/A	N/A	N/A
**Treatment completion**										
		Completed all modules	<.001	N/A	60.30	10 (9 to 11)	86.60	83.60	85.50	0.881 (0.872 to 0.891)
		Completed (4 of 5)	N/A	9.104 (7.565 to 10.956)	N/A	49 (45 to 53)	N/A	N/A	N/A	N/A
		Completed (3 of 5)	N/A	33.715 (27.454 to 41.403)	N/A	78 (75 to 81)	N/A	N/A	N/A	N/A
		Completed (2 of 5)	N/A	106.01 (79.5 to 141.36)	N/A	92 (90 to 94)	N/A	N/A	N/A	N/A
		Completed (1 of 5)	N/A	162.104 (109.23 to 240.572)	N/A	95 (92 to 96)	N/A	N/A	N/A	N/A

^a^RRI: relative risk increment.

^b^AUROC: area under receiver-operator characteristics.

^c^N/A: not applicable (redundant parameter).

^d^GAD-7: Generalized Anxiety Disorder Scale-7.

^e^PHQ-9: Patient Health Questionnaire-9.

^f^K-10: Kessler 10-Item Scale.

**Table 4:** Univariate missing cases probability replication models across the five random cross validation samples (N=6701).

Variable considered			Probability estimate of missing values at posttreatment in replication subsamples (95% CI)				
			RRI^a^ Rep^b^ 1 (n=1341)	RRI Rep 2 (n=1340)	RRI Rep 3 (n=1340)	RRI Rep 4 (n=1340)	RRI Rep 5 (n=1340)
Sample average			36 (34 to 39)	36 (34 to 39)	36 (34 to 39)	36 (34 to 39)	36 (34 to 39)
**Demographic**							
	Age (% per year)		−1.1 (−1.5 to −0.7)	−1.1 (−1.5 to −0.7)	−1.1 (−1.5 to −0.7)	−1.1 (−1.5 to −0.7)	−1.4 (−1.8 to −1)
	**Gender**						
		Female	37 (34 to 41)	36 (34 to 40)	38 (35 to 41)	37 (34 to 40)	37 (34 to 40)
		Male	33 (29 to 38)	35 (31 to 41)	31 (27 to 36)	34 (29 to 39)	33 (28 to 38)
	**Employment status**						
		At least some employment	36 (33 to 39)	35 (32 to 38)	37 (34 to 40)	36 (33 to 39)	36 (34 to 40)
		Otherwise	37 (32 to 42)	40 (35 to 45)	35 (31 to 40)	36 (32 to 42)	35 (30 to 40)
	**Relationship status**						
		In a relationship	35 (32 to 38)	34 (31 to 37)	37 (34 to 40)	35 (32 to 38)	35 (32 to 38)
		Otherwise	38 (33 to 42)	41 (36 to 45)	35 (30 to 39)	39 (35 to 44)	38 (34 to 43)
	**Education level**						
		Tertiary education	32 (29 to 36)	31 (27 to 34)	35 (31 to 39)	32 (28 to 35)	33 (30 to 37)
		Otherwise	40 (36 to 44)	41 (37 to 45)	37 (34 to 41)	40 (37 to 44)	39 (36 to 43)
**Initial severity**							
	Baseline anxiety symptoms (% per GAD-7^c^ point)		1.1 (0.3 to 1.9)	0.7 (−0.1 to 1.5)	1.4 (0.6 to 2.2)	0.4 (−0.4 to 1.2)	0.7 (−0.1 to 1.5)
	Baseline depression symptoms (% per PHQ-9^d^ point)		1.4 (0.7 to 2.1)	1.4 (0.7 to 2.1)	1.1 (0.4 to 1.8)	1.4 (0.7 to 2.2)	1.4 (0.7 to 2.1)
	Baseline psychological distress (% per K-10^e^ point)		1.1 (0.5 to 1.6)	1.1 (0.5 to 1.6)	1.4 (0.9 to 2)	1.1 (0.5 to 1.6)	1.1 (0.5 to 1.6)
	Comorbidity at baseline: (PHQ-9 ≥10 and GAD-7 ≥8)		40 (36 to 44)	40 (36 to 44)	41 (37 to 44)	40 (36 to 44)	40 (36 to 43)
	No comorbidity		33 (29 to 36)	33 (29 to 36)	32 (28 to 35)	32 (29 to 36)	33 (30 to 37)
**Treatment completion**							
	Completed all modules		9 (7 to 11)	9 (8 to 12)	11 (9 to 13)	10 (8 to 12)	9 (7 to 11)
	Completed (4 of 5)		57 (48 to 66)	47 (39 to 55)	48 (39 to 56)	51 (42 to 59)	45 (36 to 53)
	Completed (3 of 5)		77 (70 to 83)	82 (75 to 88)	72 (64 to 79)	79 (72 to 85)	81 (73 to 86)
	Completed (2 of 5)		90 (84 to 94)	91 (85 to 95)	91 (86 to 95)	95 (90 to 98)	93 (87 to 96)
	Completed (1 of 5)		95 (88 to 98)	96 (90 to 99)	95 (89 to 98)	94 (87 to 97)	93 (86 to 96)

^a^RRI: relative risk increment.

^b^Rep: randomized subsample for cross-validation purposes.

^c^GAD-7: Generalized Anxiety Disorder Scale-7.

^d^PHQ-9: Patient Health Questionnaire-9.

^e^K-10: Kessler 10-Item Scale.

### Treatment Completion

Treatment completion was measured by the progression of participants through the 5 modules of the course, consistent with definitions of treatment progression and adherence in eHealth interventions [20]. Completion was measured by (1) logging in to the assigned secured website and (2) accessing the lesson modules, either being online, when the duration of participation could be recorded, or by downloading the lessons.

### Analytical Plan

#### Identifying Predictors of Missing Cases and the Rate of Clinical Change

The characteristics of missing cases and the estimates of their likely outcomes were examined in 3 steps. All analyses were conducted using SPSS (IBM Corporation) version 25 and a dedicated *R* software package [21] for longitudinal power [22].

#### Missing Cases Probability

The first step aimed to identify the relative importance of variables that examined the probability of becoming a missing case. Testing and modeling of the probability of missing cases followed the variable selection strategy outlined by Harrell [23] for logistic regression modeling. In this strategy, potential moderating predictors were tested through separate (univariate) logistic regression models, with the missing case status of the patient at posttreatment as the binary dependent variable. Subsequently, a stepwise variable selection analysis was used to identify factors included in the multivariate model, including treatment completion; baseline depression score; baseline anxiety score; and demographic variables, such as gender, age, employment status, educational attainment, and relationship status. Variables that increased the probability of becoming a missing case were retained in the final model of predictors of missing cases probability. Additional forward and backward model building techniques were also employed to replicate the findings of the stepwise variable selection analysis. Each possible predictor of missing cases was assessed for statistical significance at a more conservative *P* value of .01. In addition, the ability of each predictor to account for the probability variance of missing cases likelihood was represented with the Nagelkerke R-squared values, which illustrates the predictive contribution of each variable and the variance it can account for in comparison with a model with no predictors [24]. The potential of each variable to differentiate between missing and nonmissing cases was evaluated with sensitivity (prediction of true positives; noncompletion), specificity (prediction of true negatives; observed), and the overall rate of prediction accuracy statistics such as receiver-operator characteristics.

#### Moderators of Clinical Change

Longitudinal statistical models were also employed to test the influence of baseline and treatment variables on the rate of symptom change. Together, these models sought to identify variables that jointly predicted missing cases and the rate of symptom change, where a significant result on both outcomes would imply a mechanism of missing cases. Longitudinal predictors of symptom change were examined using generalized estimated equation models, such as generalized estimating equations (GEEs) [25] that included a time covariate, each of the predictors as a main effect, and a time by predictor interaction. In these models, the coefficient of change between pre- and posttreatment (β_time_) represents the average rate of pre-post symptom change (longitudinal change from baseline) after accounting for within-subject variance (repeated individual scores over time). The moderation of symptom change following treatment was tested by examining the time by covariate interaction (eg, β_time*_β_Gender_). All models included a gamma scale, an unstructured pattern of within-subject correlation matrix, and a log link function to account for positive skewness and the proportional pattern of symptom change from baseline [26]. These models were also tested with the overall sample and retested within each of the 5 subsamples. The purpose of cross-replication sought to test whether characteristics of certain missing cases could be observed reliably within cross-validation subsamples.

#### Power Analyses

A power analysis was conducted for both the GEE longitudinal models of symptom change, and the binary logistic regression models of missing cases probability at posttreatment [27]. To estimate power, these analyses used the observed statistical parameters from pilot GEE models, such as the rate of change over time, the variance of symptom scores at each time point, and within-subject correlation. This information was then used to determine the minimal differences in the rate of longitudinal change (moderation of longitudinal change) that could be refuted as false negatives [22]. The pilot data used to determine the overall rate of change were replication sample 1 (n=1341), and the differences from the overall rate of symptom change, or missing cases likelihood, were calculated as the relative difference (expβ) from the overall rate of change. These power analyses determine whether nonsignificant tests of symptom change variance, or missing cases probability, are genuine nonsignificant results or whether certain nonsignificant results could be masked by the size of the sample. Separate power estimates were created for the GEE models of symptom change and the binary logistic regression models of missing cases probability. All analyses also specified the probability of power at 80% and a probability of Type I error of .05. The resulting power estimates are further described in the Results section.

#### Comparison of Different Missing Cases Outcome Approximation Models

Approximated missing cases replacement scores were generated using several types of stratified longitudinal models and evaluated side by side. Models differed from one another by the inclusion of different covariates and a covariate by a time interaction term. For example, by including covariates such as gender and a time-by-gender interaction term, the prediction of replacement outcome scores for missing cases is considered to approximate the corresponding clinical outcomes of that individual as a male or a female. The inclusion of different covariates in the models is thought to test different assumptions about why patients were missing and lead to the adjusted prediction of their likely outcomes [5,8]. In statistical terms, the conditional adjustment of missing cases outcomes by different variables is often referred to as the replacement of missing cases under a conditional missing at random assumption (MAR) [5,8].

In contrast to the adjusted models, models assumed that posttreatment missingness occurred as a completely random event. In these models, the probability of missingness was assumed to be without any systematic characteristics and was unrelated to the patient’s outcome [5,8]. These models included no individual patient covariates, other than the time coefficient, and were labeled as missing completely at random (MCAR). Under such MCAR models, the average replacement of missing cases would reflect the average outcome of the remaining sample of completers, given that missing cases are not assumed to be unique from their completer peers.

Missing cases were also replaced through statistical methods such as multiple imputations and a predictive longitudinal mixed model, which included random slopes and random intercepts [9]. The replacement outcomes from such models were used to compare the estimation of missing cases replacement across different types of statistical methods. This addition intended to establish that the impact from the phenomena of nonignorable missing case mechanisms would be observed despite different statistical techniques. Finally, the results using nonstatistical methods for missing cases replacement, such as the last observation carried forward (LOCF) method and baseline observation carried forward (BOCF) method were compared.

To gauge the accuracy and impact associated with the different replacement models, adjusted models (MAR) were compared and interpreted as either overestimating, underestimating, or being equivalent to models that overlook the features of missing cases (MCAR models). Specifically, if the mean CI from an adjusted model was within the mean CI of an unadjusted model, evidence of statistical equivalence was concluded [28]. If the CI of the mean replacement scores was outside the mean of the scores from unadjusted models cases, the models were considered to approximate distinct (statistically significant) symptom outcomes.

## Results

### Predictors of Missing Cases and the Rate of Clinical Change

Results from the logistic regression models and testing for predictors of missing cases at posttreatment are presented in Table 3.

The binary models indicated that increased psychological distress (Wald *χ*^2^_1,6701_=70.1; *P*<.001), increased baseline depressive symptoms (Wald *χ*^2^_1,6701_=152.4; *P*<.001), decreased treatment completion (Wald *χ*^2^_4,6701_=2247.4; *P*<.001), and decreased age (Wald *χ*^2^_1,6701_=183.1; *P*<.001) were significant predictors of missing cases. Together, these variables predicted 60.8% of the variance observed out of the total probability variance for becoming missing at posttreatment (Nagelkerke *R*^2^=60.80%). Predictors of missing cases included relationship status, educational attainment, and comorbidity. However, these variables accounted for a substantially lower explained variance (*R*^2^<.005) and were associated with predictive accuracy that was close to random or around 50% overall accuracy.

The effect of increased baseline severity demonstrated that for every additional PHQ-9 point at baseline, the probability of a participant becoming a missing case at posttreatment increased by 2% or 0.7% as a measure of relative risk (eg, 0.7% of 36%). Similarly, the effect of a 1-point increase in psychological distress at baseline, as measured by K-10, increased the odds of an individual becoming missing by 1.6% or 0.56% as a measure of relative risk.

The age of the participant was associated with a reduced probability of presenting as a missing case, with each additional year of age reducing the odds of becoming a missing case by 3.3% or 1.2% as a measure of relative risk. However, treatment completion, which is the number of lessons completed during treatment, was the dominant predictor of missing cases and accounted for 60.3% of the total 60.8% probability variance of missing cases. The disparity among different rates of treatment completion demonstrated that only 9.80% of participants who completed the entire program did not complete the posttreatment assessment, whereas more than 95% of those who completed only one lesson were missing cases posttreatment.

An interaction between the severity of depressive symptoms at baseline and treatment completion was found to be nonsignificant (Wald *χ*^2^_1,6701__Treatment completion*Baseline symptoms_=2.2, *P*=.71), as was the age by treatment completion interaction (Wald *χ*^2^_1,6701__Age*_ χ^2^_Treatment completion_=4.9, *P*=.30). These nonsignificant interactions imply that baseline symptom severity, age, and treatment completion were distinct predictors of missing cases probability and were independently impacting missingness (eg, additive effects that are not conditional on one another).

Table 4 provides estimates of different missing cases predictors and the replication of these results within each of the 5 subsamples.

### Power Analyses of Missing Cases Probability Models

Post hoc power analyses of the missing cases models illustrated that the 5 replication subsamples were powered to refute false-negative effects that were as little as 10% of the overall sample probability of missing cases. For example, sample 1 (n=1341) was powered to refute false-negative predictors that moderated the probability rate of missing cases by 3.6% or more (10% of the 36% who did not complete the posttreatment assessment). Refuting nonsignificant tests of predictors that were smaller than 3.6% required a sample larger than the sample available (1341). The power to refute nonsignificant results can be illustrated with the test of the gender predictor in Table 5, where missing cases of males were estimated as 33% and that of females at 37%. The difference between males and females was not statistically significant, and the sample in this study was large enough to refute this difference as a genuine nonsignificant (true negative) result, with a power of at least 80%.

**Table 5:** Longitudinal estimates of average anxiety (generalized anxiety disorder-7) symptom moderation.^a^

Characteristic			Moderation of the rate of GAD-7^b^ (anxiety) symptom change		
			*P* value	Time*Predictor interaction coefficient (exp^c^ [β]) for symptom change	Symptom change rate (95% CI)
Sample average			<.001	0.519	48 (47 to 49)
**Demographic**					
	Age (% per year)		.62	0.999	−0.1 (−0.3 to 0.2)
	**Gender**				
		Female	.29	0.975	48 (47 to 50)
		Male	N/A^d^	N/A	47 (45 to 49)
	**Employment status**				
		At least some employment	.046	0.952	49 (47 to 50)
		Otherwise	N/A	N/A	46 (44 to 49)
	**Relationship status**				
		In a relationship	<.001	0.887	50 (49 to 52)
		Otherwise	N/A	N/A	44 (41 to 46)
	**Education level**				
		Tertiary education	.46	0.984	48 (47 to 50)
		Otherwise	N/A	N/A	48 (46 to 49)
**Initial severity**					
	Baseline anxiety symptoms (% per GAD-7 point)		<.001	0.976	−2.4 (−2.9 to −2)
	Baseline depression symptoms (% per PHQ-9^e^ point)		.62	1.001	0.1 (−0.3 to 0.5)
	Baseline psychological distress (% per K-10^f^ point)		.30	1.002	0.2 (−0.1 to 0.5)
	Comorbidity at baseline: (PHQ-9 ≥10 and GAD-7 ≥8)		.09	0.963	49 (47 to 50)
	No comorbidity		N/A	N/A	47 (45 to 49)
**Treatment completion**					
	Completed all modules		<.001	N/A	49 (48 to 51)
	Completed (4 of 5)		N/A	0.82	43 (38 to 48)
	Completed (3 of 5)		N/A	0.699	35 (28 to 42)
	Completed (2 of 5)		N/A	0.694	38 (27 to 49)
	Completed (1 of 5)		N/A	0.686	40 (27 to 53)

^a^All estimated cases were derived from generalized estimating equations models and their marginal means.

^b^GAD-7: Generalized Anxiety Disorder Scale-7.

^c^exp: exponentiated.

^d^N/A: not applicable (redundant parameter).

^e^PHQ-9: Patient Health Questionnaire-9.

^f^K-10: Kessler 10-Item Scale.

### Predictors of the Rate of Clinical Improvement

Variables that moderated the rate of symptom improvement were also tested to determine whether similar variables identified to predict missingness also moderated the rate of symptom change over time. The coefficient statistics in Tables 6 and 7 illustrate the symptom change moderation, associated with each independent variable, for each of the 3 symptom outcomes, with the results presented with separate tables for depressive symptoms (Table 6), anxiety symptoms (Table 5), and psychological distress symptoms (Table 7).

**Table 6:** Longitudinal estimates of average depressive (Patient Health Questionnaire-9) symptom moderation.^a^

Characteristic			Moderation of the rate of PHQ-9^b^ (depressive) symptom change		
			*P* value	Time*Predictor interaction coefficient (exp^c^ [β]) for symptom change	Symptom change rate (95% CI)
Sample average			<.001	0.521	48 (47 to 49)
**Demographic**					
	Age (% per year)		.12	0.998	−0.2 (−0.4 to 0)
	**Gender**				
		Female	.18	0.967	48 (47 to 50)
		Male	N/A^d^	N/A	47 (44 to 49)
	**Employment status**				
		At least some employment	.02	0.946	49 (47 to 50)
		Otherwise	N/A	N/A	46 (43 to 48)
	**Relationship status**				
		In a relationship	<.001	0.893	50 (48 to 52)
		Otherwise	N/A	N/A	44 (42 to 46)
	**Education level**				
		Tertiary education	.82	0.995	48 (46 to 50)
		Otherwise	N/A	N/A	48 (46 to 50)
**Initial severity**					
	Baseline anxiety symptoms (% per GAD-7^e^ point)		<.001	1.003	0.3 (−0.1 to 0.7)
	Baseline depression symptoms (% per PHQ-9 point)		<.001	0.988	−1.2 (−1.6 to −0.9)
	Baseline psychological distress (% per K-10^f^ point)		<.001	1.003	0.3 (0 to 0.6)
	Comorbidity at baseline: (PHQ-9 ≥10 and GAD-7 ≥8)		.006	1.051	36 (34 to 37)
	No comorbidity		N/A	N/A	39 (37 to 41)
**Treatment completion**					
	Completed all lesson modules		<.001	N/A	49 (48 to 51)
	Completed (4 of 5)		N/A	0.874	42 (37 to 47)
	Completed (3 of 5)		N/A	0.779	35 (28 to 42)
	Completed (2 of 5)		N/A	0.75	33 (20 to 45)
	Completed (1 of 5)		N/A	0.711	29 (13 to 45)

^a^All estimated cases were derived from generalized estimating equations models and their marginal means.

^b^PHQ-9: Patient Health Questionnaire-9.

^c^exp: exponentiated.

^d^N/A: not applicable (redundant parameter).

^e^GAD-7: Generalized Anxiety Disorder Scale-7.

^f^K-10: Kessler 10-Item Scale.

**Table 7:** Longitudinal estimates of average psychological distress (Kessler-10) symptom moderation.^a^

Characteristic			Moderation of the rate of K-10^b^ (psychological distress) symptom change		
			*P* value	Time*Predictor interaction coefficient (exp^c^ [β]) for symptom change	Symptom change rate (95% CI)
Sample average			<.001	0.63	37 (36 to 38)
**Demographic**					
	Age (% per year)		.64	1	0 (−0.2 to 0.1)
	**Gender**				
		Female	.29	0.975	48 (47 to 50)
		Male	N/A^d^	N/A	47 (45 to 49)
	**Employment status**				
		At least some employment	.01	0.946	38 (36 to 40)
		Otherwise	N/A	N/A	34 (32 to 37)
	**Relationship status**				
		In a relationship	<.001	0.892	39 (38 to 41)
		Otherwise	N/A	N/A	32 (30 to 35)
	**Education level**				
		Tertiary education	.79	1.005	37 (35 to 39)
		Otherwise	N/A	N/A	37 (35 to 39)
**Initial severity**					
	Baseline anxiety symptoms (% per GAD-7^e^ point)		.01	1.005	0.5 (0.1 to 0.8)
	Baseline depression symptoms (% per PHQ-9^f^ point)		<.01	1.005	0.5 (0.2 to 0.8)
	Baseline psychological distress (% per K-10 point)		<.001	0.994	−0.6 (−0.9 to −0.4)
	Comorbidity at baseline: (PHQ-9 ≥10 and GAD-7 ≥8)		.08	0.962	49 (47 to 50)
	No comorbidity		N/A	N/A	47 (45 to 49)
**Treatment completion**					
	Completed all modules		<.001	N/A	38 (37 to 39)
	Completed (4 of 5)		N/A	0.881	34 (29 to 39)
	Completed (3 of 5)		N/A	0.77	27 (19 to 34)
	Completed (2 of 5)		N/A	0.763	30 (19 to 41)
	Completed (1 of 5)		N/A	0.644	18 (2 to 34)

^a^All estimated cases were derived from generalized estimating equations models and their marginal means.

^b^K-10: Kessler 10-Item Scale.

^c^exp: exponentiated.

^d^N/A: not applicable (redundant parameter).

^e^GAD-7: Generalized Anxiety Disorder Scale-7.

^f^PHQ-9: Patient Health Questionnaire-9.

Table 6 shows that posttreatment depressive symptoms were moderated by treatment completion, all 3 baseline symptom levels, and relationship status; all presenting with significant predictor by time interactions. Thus, increases in baseline symptom severity, increased treatment completion, and relationship status significantly increased the rate of depressive symptom improvement in therapy.

Significant predictors of the rate of change in anxiety symptoms were similarly identified. Specifically, increased baseline anxiety symptoms, increased treatment completion, and the relationship status in treatment seemed to increase the rate of symptom change. The results of the anxiety moderators are presented in Table 5.

Analyses exploring moderators of general psychological distress (K-10) yielded the same pattern, with the results presented in Table 7, showing treatment completion, baseline severity, and relationship status to significantly moderate changes in psychological distress.

### Power Analyses of Symptom Change Rate Models

Post hoc power analyses of the GEE symptom change models demonstrated that each of the 5 replication subsamples was adequately powered to determine which variables were nonsignificant if they moderated the rate of symptom change by as little as 12% of the total depression symptom change effect (5.7% of 48%). Within the anxiety symptom change models, the sample was powered to refute nonsignificant predictors that moderated 12% of the total reduction of anxiety symptom reduction (5.7% of 48%) and 13% of the total psychological distress symptom reduction (4.4% of 37%). Refuting predictor effects that were smaller than 5.7% (PHQ-9 and GAD-7) and 4.4% (K-10) required a sample that was larger than the 842 participants available in each of the subsamples.

### Identified Mechanisms of Nonignorable Missing Cases

The predictors of treatment completion, baseline symptoms, and, to a lesser extent, relationship status demonstrated an association with both the likelihood of missing data at posttreatment and the rate of symptom change over time. These results confirm that treatment completion and, to a lesser extent, baseline symptoms were not significantly associated with noncompletion.

**Figure 1:**
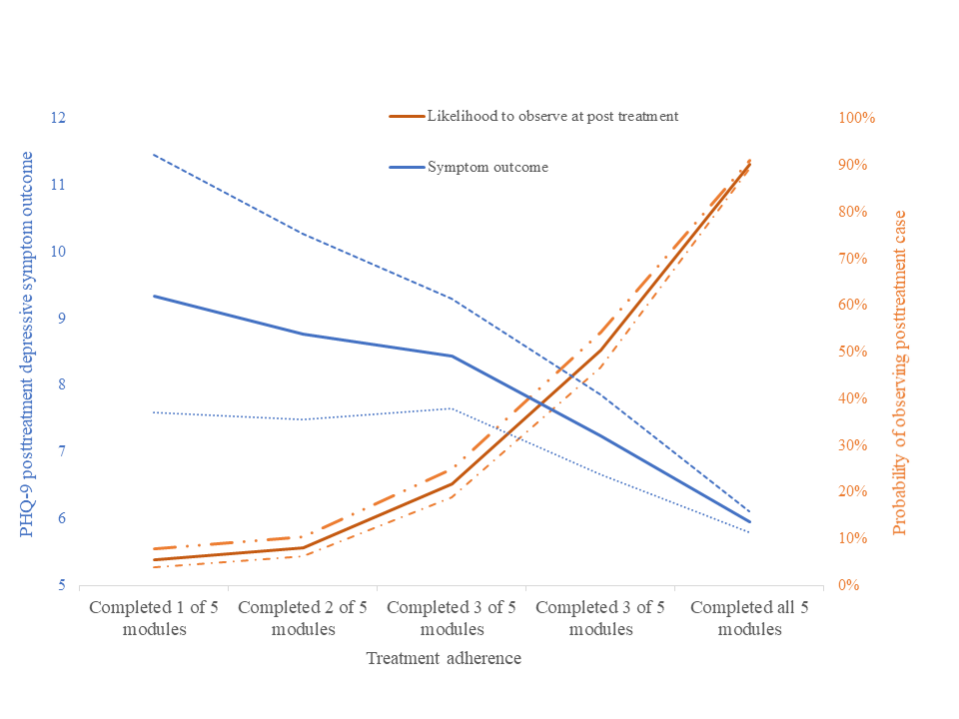
Probability for observing cases at posttreatment (inverse probability of missing cases) and treatment outcome trends associated with treatment completion; 95% CI is drawn around each effect in dotted lines. PHQ-9: Patient Health Questionnaire-9.

**Figure 2:**
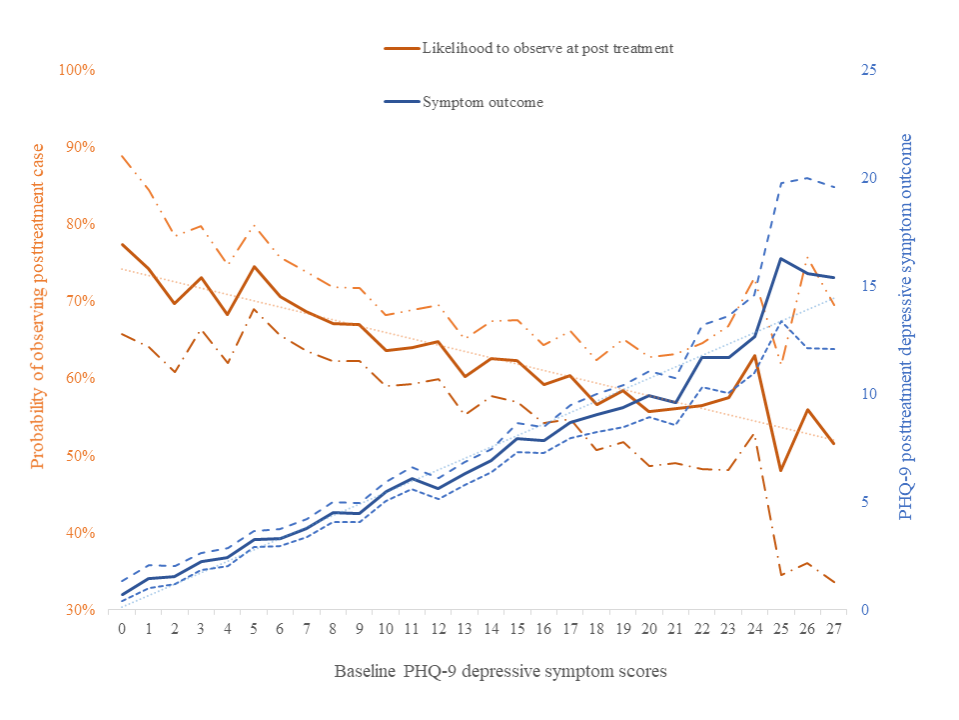
Probability for observing cases at posttreatment (inverse probability of missing cases) and treatment outcomes trends associated with depressive symptoms baseline severity; 95% CI is drawn around each effect in dotted lines. PHQ-9: Patient Health Questionnaire-9.

The association of treatment completion and baseline symptoms with both clinical improvement and risk of presenting as missing cases are illustrated in Figure 1 (missing cases probability at posttreatment and symptom change, associated with program completion) and Figure 2 (missing cases and symptom change trends associated with depressive symptom baseline severity and depressive symptom outcomes). These figures illustrate how the probability of missing cases is likely to increase for those individuals who also experience higher depressive symptoms at the end of the treatment period (8 weeks), as a result of low treatment completion (Figure 1) and increased baseline symptoms (Figure 2).

### Comparison of Replacement Outcomes From Different Statistical Models

In this step, the statistical approximation of replacement symptom outcomes was compared between 3 different statistical models: (1) models that adjust for the predictors that form missing cases mechanisms (eg, treatment completion), (2) models that adjust only for time (Completer’s analysis), and (3) models that adjust for predictors that are not considered to be a cause of missing cases (eg, gender, age, education). These models differ from one another by the inclusion of different covariates that adjust the projected outcomes of missing cases. Tables 8 to 10 present the approximated mean PHQ-9, GAD-7, and K-10 scores and the CIs for the replacement scores for the various models.

**Table 8:** Predicted Patient Health Questionnaire-9 outcomes generated with different replacement models—compared with average posttreatment model estimate (missing completely at random).

Model estimation type considered		Mean predicted posttreatment score (95% CI)	Relative to the completer case analysis (MCAR; 95% CI)	The conclusion drawn about the replacement approach
Pretreatment symptom scores from posttreatment missing cases		13.09 (12.8-13.34)	N/A^a^	N/A
Outcomes from (MCAR^b^) completer case analysis		6.3 (6.2-6.5)	N/A	N/A
**Models adjusted for predictors that do not form missing cases mechanisms**				
	(MAR^c^) Age	6.3 (6.3-6.3)	1 (1-1)	Equivalent to MCAR
	(MAR) Gender	6.3 (6.3-6.3)	0 (0-0)	Equivalent to MCAR
	(MAR) Employment status	6.3 (6.3-6.3)	0 (0-1)	Equivalent to MCAR
	(MAR) Relationship status	6.3 (6.3-6.4)	1 (0-1)	Equivalent to MCAR
	(MAR) Education level	6.3 (6.3-6.4)	1 (1-1)	Equivalent to MCAR
**Models adjusted for predictors that form nonignorable missing cases mechanisms (missingness and PHQ-9^d^ outcome moderators)**				
	(MAR) Baseline anxiety symptoms (GAD-7^e^)	6.5 (6.4-6.6)	3 (2-4)	Significant scores above MCAR
	(MAR) Baseline depressive symptoms (PHQ-9)	6.9 (6.8-7.1)	10 (8-12)	Significant scores above MCAR
	(MAR) Baseline psychological distress (K-10^f^)	6.9 (6.8-7)	10 (8-12)	Significant scores above MCAR
	(MAR) Comorbidity (PHQ-9 ≥10 and GAD-7 ≥8)	6.6 (6.5-6.6)	4 (3-6)	Significant scores above MCAR
	(MAR) Treatment adherence	8.1 (8.1-8.2)	29 (29-30)	Significant scores above MCAR
	(MAR) Treatment completion and baseline symptoms	8.8 (8.6-8.9)	39 (36-42)	Significant scores above MCAR
**Adjusted models from alternative statistical methods (missingness and PHQ-9 outcome moderators)**				
	Mixed linear model (MLM)—slopes and intercepts (adjusting for PHQ-9 baseline)	7.4 (7.2-7.6)	18 (14-21)	Significant scores above MCAR
	Multiple imputation (MI) pooling—adjusted for PHQ-9 baseline	7.6 (7.2-8)	21 (14-27)	Significant scores above MCAR
	Mixed linear model (MLM)—treatment completion, slopes, and intercepts	8.4 (8.2-8.6)	33 (30-37)	Significant scores above MCAR
	Multiple imputation (MI) pooling—treatment completion and baseline symptoms	8.8 (8.4-9.2)	40 (33-46)	Significant scores above MCAR
	Last observation carried forward (LOCF)	10.4 (10.2-10.7)	65 (62-69)	Significant scores above MCAR
	Baseline observation carried forward (BOCF)	13.1 (12.8-13.3)	108 (104-112)	Significant scores above MCAR

^a^N/A: not applicable (redundant parameter).

^b^MCAR: missing completely at random.

^c^MAR: missing at random.

^d^PHQ-9: Patient Health Questionnaire-9.

^e^GAD-7: Generalized Anxiety Disorder Scale-7.

^f^K-10: Kessler 10-Item Scale.

**Table 9:** Predicted Kessler-10 outcomes generated with different replacement models—compared with average posttreatment model estimate (missing completely at random).

Model estimation type considered		Mean predicted posttreatment score (95% CI)	Relative to Completer’s treatment effect (MCAR;^a^ 95% CI)	The conclusion drawn about the replacement approach
Pretreatment symptom scores from posttreatment missing cases		19.44 (19.1-19.8)	N/A^b^	N/A
Outcomes from (MCAR) Completer's analysis		11.4 (11.1, 11.6)	N/A	N/A
**Models adjusted for predictors that do not form missing cases mechanisms**				
	(MAR^c^) Age	11.4 (11.4-11.4)	1 (1-1)	Equivalent to MCAR
	(MAR) Gender	11.3 (11.3-11.4)	0 (0-0)	Equivalent to MCAR
	(MAR) Employment status	11.3 (11.3-11.4)	0 (0-0)	Equivalent to MCAR
	(MAR) Relationship status	11.4 (11.4-11.4)	1 (0-1)	Equivalent to MCAR
	(MAR) Education level	11.4 (11.4-11.4)	0 (0-1)	Equivalent to MCAR
**Models adjusted for predictors that form nonignorable missing cases mechanisms (missingness and K-10^d^ outcome moderators)**				
	(MAR) Baseline anxiety symptoms (GAD-7^e^)	12.4 (12.2-12.7)	10 (8-12)	Significant scores above MCAR
	(MAR) Baseline depressive symptoms (PHQ-9^f^)	12.2 (12-12.4)	7 (6-9)	Significant scores above MCAR
	(MAR) Baseline psychological distress (K-10)	11.7 (11.5-11.8)	3 (2-4)	Significant scores above MCAR
	(MAR) Comorbidity (PHQ-9≥10 and GAD-7≥8)	11.8 (11.6-11.9)	4 (3-5)	Significant scores above MCAR
	(MAR) Treatment completion	13.7 (13.7-13.8)	21 (21-22)	Significant scores above MCAR
	(MAR) Treatment completion and baseline symptoms	14.6 (14.3-14.9)	29 (26-31)	Significant scores above MCAR
**Adjusted models from alternative statistical methods (missingness and K-10 outcome moderators)**				
	Mixed linear model (MLM)—slopes and intercepts (adjusting for K-10 baseline)	12.7 (12.5-12.9)	11 (10-13)	Significant scores above MCAR
	Multiple imputation (MI) pooling—adjusting for K-10 baseline	12.1 (11.9-12.4)	6 (4-9)	Significant scores above MCAR
	Mixed linear model (MLM)—treatment completion, slopes, and intercepts	14 (13.8-14.2)	23 (21-25)	Significant scores above MCAR
	Multiple imputation (MI) pooling—treatment completion and baseline symptoms	14.1 (13.3-14.9)	24 (17-31)	Significant scores above MCAR
	Last observation carried forward (LOCF)	17.8 (17.5-18.2)	56 (54-59)	Significant scores above MCAR
	Baseline observation carried forward (BOCF)	19.4 (19.1-19.8)	71 (68-74)	Significant scores above MCAR

^a^MCAR: missing completely at random.

^b^N/A: not applicable (redundant parameter).

^c^MAR: missing at random.

^d^K-10: Kessler 10-Item Scale.

^e^GAD-7: generalized anxiety disorder scale-7.

^f^PHQ-9: patient health questionnaire-9.

**Table 10:** Predicted generalized anxiety disorder scale-7 outcomes generated with different replacement models—compared with average posttreatment model estimate (missing completely at random).

Model estimation type considered		Mean predicted posttreatment score (95% CI)	Relative to Completer's only (MCAR; 95% CI)	The conclusion drawn about the replacement approach
Pretreatment symptom scores from posttreatment missing cases		11.45 (11.2-11.7)	N/A^a^	N/A
Posttreatment outcomes from (MCAR^b^) Completer’s analysis		5.7 (5.6-5.8)	N/A	N/A
**Models adjusted for predictors that do not form missing cases mechanisms **				
	(MAR^c^) Age	5.8 (5.8-5.8)	2 (1, 2)	Equivalent to MCAR
	(MAR) Gender	5.7 (5.7-5.7)	0 (0-0)	Equivalent to MCAR
	(MAR) Employment status	5.7 (5.7-5.7)	0 (0-0)	Equivalent to MCAR
	(MAR) Relationship status	5.7 (5.7-5.7)	0 (0-1)	Equivalent to MCAR
	(MAR) Education level	5.7 (5.7-5.7)	1 (1-1)	Equivalent to MCAR
**Models adjusted for predictors that form nonignorable missing cases mechanisms (missingness and GAD-7^d^ outcome moderators) **				
	(MAR) Baseline anxiety symptoms (GAD-7)	6 (5.9-6.1)	5 (3-7)	Significant scores above MCAR
	(MAR) Baseline depressive symptoms (PHQ-9^e^)	6 (5.9-6.1)	6 (4-7)	Significant scores above MCAR
	(MAR) Baseline psychological distress (K-10^f^)	6.1 (6-6.2)	7 (6-9)	Significant scores above MCAR
	(MAR) Comorbidity (PHQ-9≥10 and GAD-7≥8)	5.9 (5.8-6)	4 (3-5)	Significant scores above MCAR
	(MAR) Treatment completion	6.8 (6.8-6.8)	19 (19-20)	Significant scores above MCAR
	(MAR) Treatment completion and baseline symptoms	7.1 (6.9-7.2)	24 (22-27)	Significant scores above MCAR
**Adjusted models from alternative statistical methods (missingness and GAD-7 outcomes)**				
	Mixed linear model (MLM)—slopes and intercepts (adjusting for GAD-7 baseline)	6.3 (6.2-6.5)	11 (9-14)	Significant scores above MCAR
	Multiple imputation (MI) pooling—adjusted for GAD-7 baseline	6.8 (6.5, 7.1)	20 (14-25)	Significant scores above MCAR
	Mixed linear model (MLM)—treatment completion, slopes, and intercepts	7 (6.8, 7.1)	23 (20-25)	Significant scores above MCAR
	Multiple imputation (MI) pooling—treatment completion and baseline symptoms	7.3 (7, 7.6)	28 (23-34)	Significant scores above MCAR
	Last observation carried forward (LOCF)	9.1 (8.8-9.3)	60 (56-63)	Significant scores above MCAR
	Baseline observation carried forward (BOCF)	11.5 (11.2-11.7)	102 (98-105)	Significant scores above MCAR

^a^N/A: not applicable (redundant parameter).

^b^MCAR: missing completely at random.

^c^MAR: missing at random.

^d^GAD-7: Generalized Anxiety Disorder Scale-7.

^e^PHQ-9: Patient Health Questionnaire-9.

^f^K-10: Kessler 10-Item Scale.

Tables 8 to 10 demonstrate that the statistical models that adjust their estimates of missing cases outcome according to the prominent characteristics of missing cases resulted in the prediction of increased symptom outcomes and a more restrained estimation of the treatment effect. For example, missing cases replacement models that account for the rate of treatment completion resulted in PHQ-9 estimates that were 29% higher than the outcomes from the Completer’s analysis (Table 8). Similarly, missing cases replacement models that adjusted for both baseline and treatment completion resulted in outcomes that were 39% higher than the average treatment effect. In contrast, the application of models that adjust missing cases replacement scores by covariates that *only* predict missing cases (eg, age) or the rate of symptom change (eg, relationship status) did not result in missing cases symptom estimates that were different than average (nonadjusted MCAR models).

The influence of nonignorable mechanisms of missing cases is repeated in Table 9 (GAD-7) and Table 10 (K-10). Accounting for the role of low treatment completion in missing cases increased the projected symptom scores for missing cases by 20%. When the role of baseline symptom severity was also included in the replacement procedure, the predicted missing cases outcomes increased to nearly 30% above the average symptom outcome scores. In contrast, models that adjust their predicted outcome by variables that do not jointly predict missing cases and symptom change have resulted in outcomes that were very close to those of the completers.

A comparison between the GEE replacement estimation, multiple imputation, and mixed model–based replacement also demonstrates that the effect of treatment completion could be reliably observed across different statistical techniques. For example, the multiple imputations and mixed model replacement methods that accounted for a measure of treatment completion (stratified) all resulted in higher and comparable symptom replacement outcomes across GEE and multiple imputation and mixed models methods and across all symptom outcomes: depression (PHQ-9), anxiety (GAD-7), and psychological distress (K-10).

Finally, LOCF and BOCF replacement methodologies were compared with the other outcomes. Tables 8 to 10 show that using BOCF and LOCF methodologies, replacement scores for missing cases were higher when compared with the statistical approximation of outcomes for completers.

## Discussion

### Principal Findings

The aim of this study is to better understand the characteristics of missing cases and compare methods for estimating the symptom outcomes of missing cases in psychotherapy. The results of the study identified the following variables: (1) treatment adherence rate, defined as the rate of module progression through a treatment protocol and (2) the severity of symptom scores before treatment as variables that moderated both the probability for a case to present as missing during posttreatment assessment and the rate of symptom reduction. Low treatment adherence in particular dominantly predicted both the odds ratio of a case to present as missing during a posttreatment evaluation (162.1:1), and at the same time, low adherence dulled the rate of symptom reduction effect by up to 29%, 41%, and 52% for anxiety, depression, and psychological distress symptoms, respectively. These results are congruent with preliminary research [4] and suggest that the effect of missing cases is fundamental for the measurement process of clinical evidence and is of vital importance to anyone interested in a complete and unbiased account of the efficacy of psychological treatment.

With regard to the hypotheses stated, the first hypothesis that treatment completion and the severity of symptoms at baseline would predict both the likelihood of missing cases and symptom outcomes was supported. Treatment completion accounted for most of the missing case probability variance at posttreatment (*R*^2^<60%). More than 95% of participants who completed all of the intervention provided symptom data posttreatment compared with the 5% of those who completed a single module. Consistent with previous research in psychotherapy, treatment completion also moderated the rate of symptom improvement for depression, anxiety, and distress, suggesting a positive dose-response relationship in the efficacy of iCBT [29,30]. Specifically, individuals who completed more of the treatment modules demonstrated up to double the rate of symptom change for psychological distress, depression, and anxiety within the same period of 8 weeks.

The identification of the association between treatment completion, noncompletion, and clinical outcomes as related concepts in a very large sample and with multiple outcomes confirms findings from earlier studies of factors associated with outcomes in psychotherapy [1,29,30]. However, in comparison, few studies of psychotherapy outcomes have examined the relationship between these variables, and instead, treatment completion, reasons for dropping out of treatment, and clinical outcomes have been defined as distinct outcomes [20] and explored as parallel outcomes in meta-analyses of noncompletion [2] or in studies of predictors of noncompletion [13].

The findings of this study are also consistent with those of previous studies [4], which suggested that noncompleters were likely to have significantly worse treatment outcomes that would be overlooked without adjusting for the rate of treatment completion and the severity of symptoms of a patient at baseline. The comparison of statistical techniques demonstrated the effect of these variables on the replacement outcomes, regardless of the statistical technique employed. For this reason, it is recommended that to produce accurate and representative replacement estimates for missing cases, researchers should account for the relationship between treatment completion, the probability of completion, and the rate of improvement of symptoms.

The key recommendation arising from these findings concerns the measurement and evaluation of treatment outcomes in both clinical trials and routine care. At present, missing case patterns are mostly overlooked [9] despite being common and comprising a substantial portion of samples examined in psychotherapy research [1]. To date, there has been comparatively little research attempting to examine the suitability of different statistical methods to handle missing cases.

The second aim of this study is to explore the suitability of different statistical solutions to replace the outcomes of missing cases and identify methodological opportunities for psychotherapy researchers. From the range of patient characteristics, 2 types of models were identified: (1) models that included the key nonignorable mechanisms of treatment completion and (2) models that included alternative less dominant predictors, such as age, gender, and education. For example, the analyses of psychotherapy patient characteristics demonstrated that higher psychological distress symptoms at baseline, higher depressive symptoms at baseline, or relatively younger age, also predicted the increased probability of noncompletion. This study found that age, gender, and baseline symptoms are limited in their ability to account for the variance in missing cases (*R*^2^<5%) or account for the outcomes of missing cases. In contrast, treatment completion far outweighed other competing explanations for missing cases. In this manner, the study results supported the second hypothesis postulating that models that adjust for treatment completion and baseline severity would be more representative of the outcomes of missing cases.

In technical statistical terms, the joint association of the treatment adherence variable with missingness probability and the rate of symptom change is considered to demonstrate a nonignorable mechanism of missing cases. Simply put, the results show that missing cases do not occur as a random event and that missing cases outcomes do not compare with the remaining sample. This study, together with previous research [4], demonstrated that the inclusion of a single key treatment adherence covariate is enough to substantially improve the prediction and replacement of missing cases outcomes. Such findings support the proposed recommendation to use treatment completion as a key mechanism of missing cases and as an adjustment variable in the process of approximating missing cases outcomes [5,31].

### Limitations and Future Directions

The findings must be considered in light of several key limitations. First, the demonstration of missing cases, their characteristics and outcomes, and the suitability of replacing missing cases through adjusted models can only be considered preliminary and, at this time, relevant to iCBT [15]. Given that missing cases estimates vary between treatments [2,9], it is possible that the patterns, predictors, and outcomes of missing cases also vary between treatment models. Although this sample employed extensive cross-validation efforts, the trajectories of missing cases identified in this sample should be considered preliminary and experimental. Replication of these findings using different treatments could affirm the generalizability of early treatment completion as a key mechanism of missing cases and the importance of treatment completion for clinical improvement in psychotherapy. Specifically, additional and more detailed replications of the findings across different clinical contexts, such as trials with differing outcome measurement methodologies (eg, self-reported vs clinical diagnosis [32]), differing levels of treatment intensity [30], and differing timelines within study methodology [33], are needed to further verify the validity of treatment adherence as a mechanism that shapes the prediction of missing cases outcomes in psychotherapy research.

Second, this study was unable to examine other variables influencing the trajectories of missing cases or test all of the theoretical causes of missing cases, for example, the effect of interaction between a participant and an individual therapist despite the regimented nature of iCBT or the intervention of external events affecting participation. Other possible variables include the presence of major depression [32,34], perception of treatment credibility [35], or motivation [13] that can also affect treatment completion and the trajectory of participants in psychotherapy. Future studies may consider a more direct or more sophisticated measurement of participant engagement, such as motivation and time spent engaged with treatment, and even directed follow-up surveying to explore why patients dropped out of treatment and lapse out of the assessment protocol.

In addition, although not a limitation of this study, it is important to note that the ability to use statistical replacement models adjusted by treatment completion and baseline symptoms may not be realistic in studies involving small samples [27], where many psychotherapy trials involve samples less than 50 patients and do not have the statistical power to confirm the associations found in this study. In smaller studies, LOCF for cases that do not complete treatment (eg, less than 80% adherence) could be combined with the replacement values from unadjusted models for cases who complete treatment in full (MCAR). Such an approach could result in a less statistically demanding procedure that balances overly conservative LOCF statistics with overly liberal unadjusted model approximation [1].

In conclusion, this study aimed to explore the characteristics of missing cases, the possible clinical outcomes of missing cases in internet-delivered psychotherapy, and the suitability of different strategies for accounting for the outcomes of missing cases in psychotherapy trials. The findings of this study suggest that (1) missing cases are associated with lower treatment completion, (2) the clinical trajectories of missing cases are not likely to be similar to the average participant, and (3) overlooking the nonignorable mechanisms of missing cases is likely to result in erroneous replacement of missing cases outcomes and inflated estimates of treatment effects. The findings suggest that researchers need to consider how they account for the outcomes of missing cases in psychotherapy trials where nonignorable missing cases mechanisms are likely to occur. Accounting for missing cases in this manner provides a more realistic estimate of treatment effects in the *real world*, as it is expected that some participants will drop out. In this manner, more complete and realistic estimates that account for the outcomes of missing cases can contribute toward more realistic psychotherapy evaluation and outcome modeling.

## Abbreviations

BOCF: baseline observation carried forward
DMHS: digital mental health service
exp: exponentiated coefficient
GAD-7: Generalized Anxiety Disorder Scale-7
iCBT: internet-delivered cognitive behavior therapy
K-10: Kessler 10-Item Scale
LOCF: last observation carried forward
MAR: missing at random
MCAR: missing completely at random
PHQ-9: Patient Health Questionnaire-9
